# Translating global recommendations on HIV and infant feeding to the local context: the development of culturally sensitive counselling tools in the Kilimanjaro Region, Tanzania

**DOI:** 10.1186/1748-5908-1-22

**Published:** 2006-10-03

**Authors:** Sebalda C Leshabari, Peggy Koniz-Booher, Anne N Åstrøm, Marina M de Paoli, Karen M Moland

**Affiliations:** 1 University of Bergen, Center for International Health, Norway; 2 Muhimbili University College of Health Sciences, School of Nursing, Tanzania; 3 University Research Co., LLC, Quality Assurance Project, USA; 4 Fafo Institute for Applied International Health, Norway; 5 Bergen University College, Norway

## Abstract

**Background:**

This paper describes the process used to develop an integrated set of culturally sensitive, evidence-based counselling tools (job aids) by using qualitative participatory research. The aim of the intervention was to contribute to improving infant feeding counselling services for HIV positive women in the Kilimanjaro Region of Tanzania.

**Methods:**

Formative research using a combination of qualitative methods preceded the development of the intervention and mapped existing practices, perceptions and attitudes towards HIV and infant feeding (HIV/IF) among mothers, counsellors and community members. Intervention Mapping (IM) protocol guided the development of the overall intervention strategy. Theories of behaviour change, a review of the international HIV/IF guidelines and formative research findings contributed to the definition of performance and learning objectives. Key communication messages and colourful graphic illustrations related to infant feeding in the context of HIV were then developed and/or adapted from existing generic materials. Draft materials were field tested with intended audiences and subjected to stakeholder technical review.

**Results:**

An integrated set of infant feeding counselling tools, referred to as 'job aids', was developed and included brochures on feeding methods that were found to be socially and culturally acceptable, a Question and Answer Guide for counsellors, a counselling card on the risk of transmission of HIV, and an infant feeding toolbox for demonstration. Each brochure describes the steps to ensure safer infant feeding using simple language and images based on local ideas and resources. The brochures are meant to serve as both a reference material during infant feeding counselling in the ongoing prevention of mother to child transmission (pMTCT) of HIV programme and as take home material for the mother.

**Conclusion:**

The study underscores the importance of formative research and a systematic theory based approach to developing an intervention aimed at improving counselling and changing customary feeding practices. The identification of perceived barriers and facilitators for change contributed to developing the key counselling messages and graphics, reflecting the socio-economic reality, cultural beliefs and norms of mothers and their significant others.

## Background

The documentation of breastfeeding as a source of human immunodeficiency virus (HIV) infection in babies born to HIV positive mothers represents a public health dilemma, especially in countries with a high HIV prevalence rate and where breastfeeding is the norm and essential to child survival [1-4]. According to the UNAIDS update for 2005, 700,000 infants are HIV infected every year, with an estimated 5 to 15 percent of children born to HIV positive women being infected through their mother's milk [5]. As knowledge about the risk of HIV transmission through breastfeeding has reached health care workers, the general population, and individual mothers, uncertainty has developed on how best to feed infants in the context of HIV. Women who know or suspect they are HIV positive are faced with difficult and complex choices [6].

Current international guidelines [2] on infant feeding for HIV positive mothers promote *replacement feeding *(infant formula or animal milk) or *exclusive breastfeeding *(with no supplements of any kind). A mixed feeding pattern, where breastfeeding is combined with other milks, liquid foods or solids, has been shown to increase the risk of transmission [7-9] and is strongly discouraged. Current guidelines state: 'When replacement feeding is *not *acceptable, feasible, affordable, sustainable and safe (AFASS), exclusive breastfeeding is recommended during the first months of life' [2]. Based on the principle of *informed choice*, health workers are encouraged to give HIV infected women the best available information on the risks and benefits of each feeding method, with *'specific guidance in selecting the option most likely to be suitable for their situation' *[2].

Prevention of Mother To Child Transmission (pMTCT) programmes are rapidly expanding throughout sub-Saharan Africa, with several key intervention pillars: voluntary counselling and testing (VCT), anti-retroviral  prophylaxis and infant feeding counselling [10]. However, inadequate training of health workers, particularly pMTCT counsellors, related to the relative risks associated with infant feeding in the context of HIV, the feasibility and safety of replacement feeding, lack of culturally sensitive counselling tools and the stigma associated with both replacement feeding and exclusive breastfeeding make appropriate and effective infant feeding counselling difficult [7]. According to previous research, mothers' adoption of and adherence to the recommended feeding methods is also a problem [11-13]. A study in Nairobi, Kenya, that aimed to determine feeding practices and the nutritional status of infants born to HIV-1 infected women, for example, reported that 31% of the HIV positive, counselled mothers participating in the study practised mixed feeding six weeks after delivery [14]. One of the major challenges facing women in adopting and adhering to current recommendations is access to good quality information [15]. Research shows that many counsellors are not adequately informed about how to protect infants from HIV transmission and may not even be aware of the existence of updated guidelines [6,11]. Few have received sufficient training on counselling in the context of HIV [16], and pMTCT programmes in general lack counselling tools and other resources [17]. Staff shortages and the associated lack of time to counsel properly, even for those adequately trained in infant feeding counselling are further barriers to the provision of informed infant feeding choices [18].

This article describes the development of an integrated set of counselling tools, referred to as 'job aids', based on the updated international guidelines and related World Health Organization (WHO) and UNICEF generic counselling materials. The development process followed an intervention mapping (IM) framework [19], with the ultimate aim of producing a cost-effective, culturally sensitive and technologically appropriate set of tools to improve the quality and relevance of infant feeding counselling. A further objective was to strengthen HIV positive mothers' ability to both make an informed choice and safely execute a feeding method appropriate to their personal situation.

Job aids have gained status in health promotion as a cost-effective way to improve the *performance *of service providers, such as nurses, and are often defined as tools that reduce guesswork, minimize reliance on memory and promote compliance with standards [20,21]. Decision aids, or client oriented job aids, are often used to guide patients through a series of steps, giving them personalized information and/or helping them clarify their values and risk exposure in the context of health related options [20,22]. Job aids often feature visual images or graphics to enhance users' understanding of written information. To strengthen the relevance and potential for identification, both the images and the written messages should resonate with people's beliefs. In the development of the job aids reported here, both written messages and visual images were developed to reflect the local social and cultural context in the communities.

The study was located at the pMTCT clinic at KCMC (Kilimanjaro Christian Medical Centre) outside Moshi town in Kilimanjaro Region in northern Tanzania, where the HIV prevalence rate in the antenatal population is estimated at 5.7% [23]. Breastfeeding is normative in the area, but early supplementation with water, cow's milk and porridge ('partial' or 'mixed' breastfeeding) is standard practice [11]. The pMTCT clinic at KCMC recruits patients from the antenatal clinic, which primarily serves women from Moshi town and its rural outskirts. It offers the standard package of VCT, ARV prophylactics and infant feeding counselling to pregnant women and their partners.

## Methods

### Use of intervention mapping (IM) in the planning process

The importance of careful theory based intervention planning has been recognized since the publication of the Precede-Proceed model [24], where a needs assessment is conducted to identify the health problems to be addressed, the health behaviours that should change, and the psychosocial and environmental determinants to be translated into interventions. Building on the needs assessments, IM uses a stepwise approach in developing programme objectives (i.e., performance and learning objectives) and guiding the selection of intervention strategies and intervention tools [19,25]. IM promotes close collaboration between programme developers, the target population and programme users, increasing the probability of developing a user relevant intervention. IM suggests five steps based on established theories, empirical evidence and additional qualitative and quantitative research [19]. This study addresses IM steps 1 to 3.

IM Step 1 is to define the performance objectives or the behaviours that need to be taught to achieve the overall aim of the intervention programme. In turn, learning objectives are specified (e.g. mothers recognizing the importance of exclusive breastfeeding) based on the individual and environmental determinants (e.g. awareness, attitudes, social support and self-efficacy) of those performance objectives (exclusive breastfeeding). For mothers to accomplish behaviour change related to breastfeeding, recognizing the importance of that behaviour (attitudes) and utilizing external sources (social support) and personal skills to cope with barriers (self-efficacy) might be important learning objectives. Potential individual and environmental determinants of recommended practices were identified from literature reviews, focus group discussions (FGDs) as well as reviews of theoretical models [19]. The learning objectives specified were thus intended to answer the question: "What does the target group need to learn about a specific behavioural determinant in order to accomplish the performance objectives?"

Step 2 of IM uses theory as a foundation for selecting educational methods and strategies that match the learning objectives. Bandura's Social Cognitive Theory (SCT) provides a framework for articulating learning objectives, combining individual and social factors that influence practices. In accordance with SCT, it was postulated that 1) mothers who have inadequate *knowledge *about mother to child transmission of HIV would not decide to change their infant feeding practice, 2) mothers who consider their baby to be constantly *at risk of HIV infection *will be hampered in their decision to change their feeding method, 3) mothers who perceive serious *disadvantages *associated with recommended feeding methods would not change existing feeding habits, 4) mothers whose *significant others *(e.g. husbands and/or mothers in law) insist on a mixed feeding pattern will not easily choose or adhere to exclusive breastfeeding and 5) mothers who lack *confidence *in their ability to carry out a recommended feeding method may end up feeding their infants in a customary manner. Following the SCT [26], specific techniques that include information transfer, role modelling, skill building, social support and reinforcement have been developed to modify self-efficacy and other beliefs. These techniques have been widely applied and found to generate behaviour change [19,27]. These selected educational methods were further translated into practical strategies and key messages. Step 3 of IM is to develop the programme and to pre-test materials which are the major focus of this paper. Step 4 and 5 consist of programme adoption, implementation and evaluation, which will be discussed in a subsequent paper.

### Using a participatory approach

Strategic participation and consensus building between all major stakeholders was seen as critical to the process of developing the intervention, in order to ensure its social and cultural relevance and scale-up. Policy makers, technical experts, service providers and clients were involved in various phases of the process. HIV positive mothers, local community members and nurse counsellors responsible for the day to day running of the pMTCT programme participated in the formative research and in the field testing of draft materials. Members of the national consultative group responsible for developing guidelines on human immunodeficiency virus and infant feeding (HIV/IF) and other national and international technical experts provided technical guidance during the planning process as well as during the materials' design/adaptation of technical content and images from existing generic materials. A broad participation in the technical review of draft materials was achieved through electronic correspondence and the simultaneous transfer of digital graphic files to reviewers via the internet.

### Formative research

The study team conducted formative research between August 2003 and February 2004 with a double purpose: 1) to identify existing, strongly held beliefs and behaviours to be addressed by the intervention, and 2) to determine how to effectively communicate these messages in a culturally appropriate and relevant manner through key messages and illustrations. All discussions and interviews were conducted in Swahili (Tanzania's national language) using interview/discussion guides and were tape recorded, transcribed and translated into English.

With the assistance of community leaders, the team conducted 15 interviews with key informants: traditional birth attendants, community elders, members of community health committees and nurse counsellors. Eight focus group discussions (FGDs), each with 8–12 participants, were conducted among 'ordinary' community members in two wards in Moshi District. The aim was to assess knowledge, beliefs and attitudes about pMTCT, breastfeeding, replacement feeding, mixed feeding and safe sex. In order to promote homogeneity and active participation, participants were recruited by age and gender (young women, older women, young men and older men). Ten HIV positive mothers who were recruited through the pMTCT programme at KCMC, and who gave their consent to participating in the study, were visited at home and interviewed about their views of and experience with infant feeding. In order not to raise suspicion and cause involuntary disclosure of HIV positive status, other post-natal mothers were also visited in their homes and interviewed on infant feeding.

### Field testing of illustrations and draft materials

As part of the intervention, the study team aimed to develop culturally appropriate images for the job aids that reflected the local environment, dress code and ideals related to family life and infant feeding. Digital photographs were taken in homes and communities for use as references for the development of high quality, colourful illustrations using a state of the art computer graphics technique. This process allowed images to be easily altered based on feedback from both communities and technical subject experts. Initial drafts of the illustrations were pilot tested in four FGDs composed of mothers and community members in different villages on the outskirts of Moshi town, as well as among pMTCT counsellors working at KCMC. A colour copy of each image was laminated for circulation during FGDs to elicit participants' feedback on the colours and other aspects of the images. FGD participants received black and white photocopies of all images to hold and study during the group session. This field testing process was critical to the finalization of the initial set of materials in that: 1) it provided essential feedback from community members and the counsellors that enhanced the overall quality and acceptability of the images; and 2) underscored the important role of the illustrations in communicating key messages visually. Based on the field test results, adjustments to the illustrations were made, including the relative sizes of the infants, colours and type of clothing, composition of cooking fires and utensils used for preparing replacement feeds.

### Simulated counselling sessions

Finally, the research team observed nurse counsellors during simulated counselling sessions with mothers where different infant feeding options were discussed. Simulation was necessary given institutional restrictions on direct observation of counselling and provided important insights into standard client provider interaction and counselling practices.

### Data collection and analysis of data

Interviews, FGDs and observations were conducted by the first author (native to the area), with the support of an experienced local female research assistant. A local elder arranged the interviews and FGDs at community level. Great care was taken to ensure that all the information collected remained confidential. The counselling tools were field tested and modified before final production. The analysis was performed using the 'thematic content analysis' frameworks [28,29], consisting of reading and re-reading the field notes and transcribed texts, manual coding in the margins, and synthesizing and grouping data in relatively exhaustive categories.

### Ethical permission

National, regional and local authorities in Tanzania, including the Tanzania National AIDS Control Programme, the medical authorities in the Kilimanjaro region and the ethical committee at KCMC provided approval to conduct the research. Each participant provided informed consent to participate.

## Results

### Perceived risk of mother to child transmission of HIV (MTCT)

Focus group participants understood that infants can be infected with HIV through their mothers during pregnancy, delivery and breastfeeding, but the relative risk of transmission was strongly overestimated. The common belief was that if a mother is HIV positive, her infant will be automatically infected. Although the HIV positive women who had been counselled were generally better informed about MTCT than the focus group participants, they also overestimated the risk and underestimated the potential of prevention through safer infant feeding and safe sex during breastfeeding.

### Knowledge, practices and beliefs associated with HIV/IF options

#### Exclusive breastfeeding

All focus groups saw breastfeeding as the best way to feed an infant and believed it should preferably be practised into the second or third year of life. Exclusive breastfeeding, however, was not seen as being customary or feasible beyond three months because breast milk was considered insufficient for the child's growth and because mothers generally had to resume activities outside the house (FGDs and interviews). Poor maternal nutrition was also mentioned as an obstacle (interviews). There was a common belief that babies need water in their first month because they 'feel thirsty', and FGDs reported that sometimes babies were given water even before breastfeeding was established. Boiled water and gripe water were seen as essential for the relief of abdominal colic, and many believed that water should be given at least daily. Complementary foods were usually introduced before the baby reached three months (FGDs and interviews). Interviewed mothers reported that they introduced light porridge mixed with cow's milk at around two months because they believed their milk was not enough to make the baby grow 'fat and shiny' as expected by kin and neighbours. Mothers were generally concerned that exclusive breastfeeding might raise suspicion of HIV positive status.

#### Cow's milk feeding

Cow's milk, usually diluted with water and sugar, was the feeding method most commonly used as a supplement to breastfeeding (FGDs and interviews). However, it was not generally regarded as an adequate replacement for breast milk unless the mother had died or had very good health reasons for not breastfeeding (all FGDs).

#### Commercial infant formula

FGDs indicated that infant formula was not considered the best way to feed an infant and was too expensive for most people. Mothers interviewed reported that they were generally uncertain about the use of infant formula, and those who had used it experienced problems calculating the right amounts of formula powder and water. Opinions on the use of leftover formula were divided: many of the FGD participants were concerned that formula should not be discarded, but mothers who had been counselled said that leftover formula should be. Some mothers reported that for convenience they prepared the formula once a day and kept it in a thermos from morning to evening.

#### Other animal milks

Although the updated international guidelines and generic counselling materials provide guidance on preparing other animal milks as breast milk replacement (e.g. goat, camel, evaporated cow's milk and powdered whole cow's milk), the formative research revealed that these alternatives were generally not available or prohibitively expensive in the Kilimanjaro markets.

#### Expression and heat treatment of breast milk

The feasibility and acceptability of expressed and heat treated breast milk was also discussed during focus groups and interviews. Community participants stated that this option seemed too time consuming to be a practical alternative to breastfeeding. Several mentioned that expression of breast milk was strongly associated with stillbirths, infant deaths or pre-term births (FGDs and interviews). Nurse counsellor 'informants' mentioned, however, that hospital staff used to teach hand expression as part of normal breastfeeding counselling under the Baby Friendly Hospital Initiative in the 1990s, and some agreed that it was important to provide information to mothers on this technique. The concept of heating expressed breast milk, however, was strongly rejected by a number of participants.

#### Wet nursing

Focus group participants reported that wet nursing by a close relative, such as a grandmother or an aunt, used to be an alternative for orphans and infants born to sick mothers. However, due to fear of HIV transmission, wet nursing is no longer considered safe and has been discontinued. Mothers reported that they would not consider wet nursing because it would encourage neighbours and kin to ask questions on ones HIV status.

### Perceived disadvantages of replacement feeding and exclusive breastfeeding

Apart from the practical and economic disadvantages of replacement feeding, the focus group participants were concerned that a mother who did not breastfeed her infant would jeopardize her reputation as a 'good mother'. People would suspect that she had a lover or that she was HIV positive. Mothers explained that community commitment to breastfeeding is so culturally embedded that refusal to breastfeed, without a strong reason, could result in loss of respect, rejection and withdrawal of the assets otherwise granted to a woman during postnatal confinement. Both *not *breastfeeding and a baby's failure to thrive are increasingly associated with maternal HIV infection (FGDs). At the same time, exclusive breastfeeding beyond two or three months, the 'normal' period, without giving any supplements could also be interpreted as an indication that the mother might be HIV positive (interviews).

### Experiences of social pressure and lack of control

Although all HIV positive mothers who had been counselled perceived replacement feeding as the best option in terms of MTCT risk reduction, most ended up breastfeeding, some after initially opting for and/or initiating replacement feeding. They explained that they could not withstand the social pressure to breastfeed and were concerned about their reputation as good mothers. They were aware that they should either exclusively breastfeed or exclusively replacement feed to reduce the risk of MTCT, but they all perceived these methods as difficult since they could not fully control the feeding situation. FGDs revealed that mothers in law have considerable power in issues related to infant feeding. Women who spent the confinement period in their mother in law's house all felt that they had to breastfeed while also experiencing great problems preventing the mother in law from giving water and other supplements to the baby, often within the first few days or weeks of birth.

Some mothers reported giving their babies additional fluids and foods to save their own energy. One mother said during an interview: "I would rather mix feed the baby than have people pointing fingers at me, whispering behind my back that my body looks thin and that I was probably HIV infected."

### Lack of knowledge and confidence in implementing the recommended feeding options

Mothers who had been counselled reported that it was difficult to understand the advantages of exclusive breastfeeding compared to mixed feeding, and that exclusive breastfeeding was hard to practise. They reported that they did not feel adequately informed about HIV/IF and that the information was often given on the same day that they received their HIV test results. Only two out of ten HIV positive mothers interviewed could recall HIV/IF information from the counselling session. Mothers who chose replacement feeding after being counselled expressed uncertainty about preparing the formula or cow's milk, especially calculating feeding quantities and frequency. None received written instructions to take home. Mothers who chose breastfeeding reported receiving little or no guidance on exclusive breastfeeding or breast care. Problems with breastfeeding included uncertainty about how to manage cracked or bleeding nipples and thrush in the baby's mouth. The experience of painful, hot and engorged breasts was confirmed as a major cause for discontinuing breastfeeding. Poor positioning of the baby during breastfeeding was observed during home visits.

### Nurse counsellors' knowledge, practices, perceptions and beliefs

pMTCT nurse counsellors reported that they found it difficult to promote exclusive breastfeeding as an option since they did not believe that mothers could or would adhere to this method, for a variety of reasons, especially for more than two or three months after birth. Many believed that replacement feeding, and in particular infant formula, was the best option for preventing MTCT and generally recommended this feeding method, even if they did not think it was feasible. They reported that the major barrier to commercial formula feeding was cost. Very few referred to gender or other contextual issues such as poor decision making power on the part of the woman, fear of disclosure, or social pressure to breastfeed. Literacy and access to clean water and fuel needed for safe formula feeding were not mentioned as conditions affecting which feeding method(s) to recommend.

The counselling simulation revealed that the counselling session was constructed as a traditional client provider situation [30], where the nurse counsellor informed the client about the different feeding options but actually gave 'strong advice' on which to choose. A supportive dialogue was not established, practical guidance was absent, and the time spent with each mother was considered inadequate.

The formative research process revealed a high level of consensus among the different stakeholders concerning infant feeding, infant feeding in the context of HIV, and the appropriateness of the various feeding methods.

## Discussion

The formative research findings underscore the complexity of HIV/IF and associated pMTCT counselling. Problems include counsellors and the individual clients' knowledge, the mother's decision making power, collective infant feeding norms and beliefs, poor access to information and resources (counselling tools and take home materials), time constraints and limited inter-personal communication and counselling skills.

### Dissemination of findings and initial consensus building

In line with the study team's participatory approach, the formative research findings were disseminated and subsequently discussed with different groups of stakeholders at facility, district, regional, national and international level. Both electronic correspondence and face to face meetings were used to achieve the broadest possible participation of various national and international stakeholders and other technical experts. These discussions aimed to disseminate information on the barriers and facilitators of change of infant feeding, to develop a feasible behaviour change *strategy *and to obtain consensus and support for the proposed intervention.

### Rationale for the focus on developing an integrated set of job aids

In selecting the intervention strategy, a number of important issues were taken into consideration, including time for developing the intervention, available resources and infrastructure. The study team fully realized that not all aspects of this very complex, multi-dimensional problem could be addressed with just one intervention. Given that the pMTCT programme was already well established at KCMC, that a relatively high level of trust was enjoyed by the pMTCT nurse counsellors and that the government planned to scale up its pMTCT programme, the idea of strengthening pMTCT counselling services was determined to be the most appropriate focus. Although the study team recognized that a health systems approach may have limited impact in a context where infant feeding decisions are traditionally made at home, the formative research confirmed that ideas emanating from the health care system generally reach the larger population. The pMTCT programme was acknowledged as the major arena for information exchange related to infant feeding in the context of HIV counselling and testing.

### Development of performance and learning objectives and key messages

Following dissemination of findings and initial consensus building, performance objectives were identified for the HIV positive mother – to either exclusively breastfeed for up to six months or exclusively replacement feed. Performance objectives were also identified for the counsellors – to practise culturally sensitive counselling based on the updated international HIV/IF guidelines, and to use AFASS criteria for assisting HIV positive mothers in selecting the most appropriate infant feeding method based on their own personal situation. Based on the formative research and guided by the IM protocol, personal and social determinants of the recommended feeding methods were articulated (e.g., perceived risk, knowledge and beliefs, perceived social and practical disadvantages) and were matched with educational strategies, key messages and visual images. Table 1 and 2 list the learning objectives and their modifiable determinants with related educational strategies for mothers and counsellors that were applied during the development of the intervention. Drafts of WHO and UNICEF generic counselling materials were collected along with other existing infant feeding related counselling and information, education and communication (IEC) materials. Existing materials were reviewed as part of a benchmarking process, and their appropriateness was assessed in light of the formative research findings, the established learning objectives and feedback from stakeholders and other technical experts. Local adaptations of the technical content of specific generic infant feeding materials were proposed by the study team based on the key messages that were determined to be culturally and socially acceptable/relevant. Ideas were also identified through the formative research for developing and/or adapting images.

**Table 1:** Selected educational methods and strategies related to learning objectives and modifiable behavioural determinants among breastfeeding mothers

**Performance objective: ***Exclusive breastfeeding*	**Modifiable behavioural determinants**			
**Learning objectives**	**Awareness-attitudes**	**Preferences**	**Self efficacy/skills**	**Social influence**

Mothers can explain positive health consequences for the baby following exclusive breastfeeding and giving colostrum	Information transfer, presenting personally relevant information, content and images of breastfeeding brochure	Information transfer, personally relevant information, content and image of brochure		

Mothers have confidence and can practice proper positioning of baby at the breast when breastfeeding	Information transfer, presenting personally relevant information, content and images of breastfeeding brochure		Instruction – presenting topics in recognizable situations, showing techniques, facilitating factors-content and images of brochure	Role modelling, content and images of take home brochures

Mothers can name important persons to consult in case of breast problems	Information transfer, presenting personally relevant information, content of and images of breastfeeding brochure		Encouragement to ask for help and assistance, facilitating factors, content and images of brochure	

Mothers can explain positive health consequences of safe sex	Information transfer, presenting personally relevant information, content and images of brochures, counselling card	Information transfer, linking new information to old		

Mothers will have adequate perception of incidence and prevalence of MTCT	Information transfer, presenting personally relevant information, counselling card			

Mothers can explain what she can do in case of breast problems	Information transfer, presenting personally relevant information, content and images of breastfeeding brochure	Personally relevant information	Instruction-practices, positive reinforcement, discuss how to overcome barriers	Role modelling-image of brochures

**Table 2:** Selected educational methods and strategies related to learning objectives and modifiable behavioural determinants among pMTCT counsellors

**Performance objective: ***Counselling on infant feeding options*	**Modifiable behavioural determinants**			
**Learning objectives**	**Awareness-attitudes**	**Preferences**	**Self efficacy/skills**	**Social influence**

Good interpersonal relationship with mothers	Information transfer-training, Q&A Guide, content and images of brochures, counselling card		Instruction on how to overcome barriers, training interpersonal communication	

Has confidence with respect to counselling mothers on exclusive breastfeeding in the context of HIV	Information transfer, training, Q&A Guide, content and images of breastfeeding brochure, counselling card		Instruction on how to overcome barriers, facilitating factors, training interpersonal communication	Receive information about family attitude and behaviours

Can explain to the HIV infected mother how to negotiate replacement feeding	Information transfer, training, Q&A Guide, content and images of replacement feeding brochures, counselling card		Training interpersonal communication, feedback, positive reinforcement, role modelling	Information on the attitudes and behaviours at home and in the community

### Technical content and illustrations used in the job aids

The job aids developed in this study were designed to support infant feeding counselling in ongoing pMTCT programmes and infant feeding practices by mothers in their home environment. They were meant to be reviewed with clients during a counselling session to strengthen and improve counselling, increase knowledge transfer, encourage informed choice and reinforce positive behaviour change. They were then intended to be given to the client to take home as a personal reference or memory aid to support adherence to the recommended infant feeding methods.

During the development/adaptation process, the study team sought to present the basic, essential information using a logical sequence (flow) of key messages and high quality graphics. The text was developed initially in English to facilitate a broad participatory technical review, and subsequently translated into the local vernacular, Swahili. The content targeted the literacy level and socio-cultural values of the local communities. Since educational levels in the region are relatively high, fairly large amounts of text were allowed. To ensure a minimum comprehension, however, colourful graphic illustrations reflecting the cultural characteristics and clothing, typical family life and locally available technologies (e.g. utensils and cooking fires) were selected to visually support and communicate the major technical content (key messages). The illustrations, considered an essential element of the job aids, highlight images of mothers safely feeding infants following the recommended HIV/IF guidelines.

### Description of each element of the integrated set of job aids

The integrated set of HIV/IF job aids included a Question and Answer Guide (Q&A), infant feeding method brochures, a counselling card on the relative risks of HIV infection and an infant feeding 'tool box'.

#### The Question & Answer Guide (Q&A)

**Figure 1:**
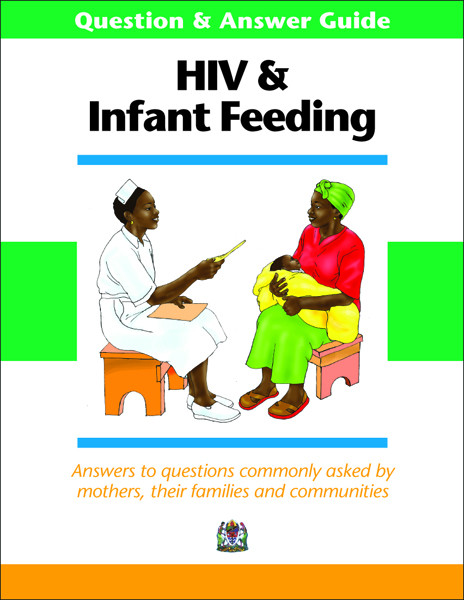
Shows Question & Answer Guide for counsellors on commonly asked questions about HIV and infant feeding.

The Q&A was designed for use during training and as a reference for health care workers to help answer commonly asked questions about HIV and infant feeding. It summarised the current international guidance on HIV/IF in a simple to read and graphically illustrated question and answer format. Questions were divided into four categories: protecting babies from HIV; infant feeding options; advantages and disadvantages of the most popular options; and safer breastfeeding and maternal nutrition. (See Figure 1.)

#### The exclusive breastfeeding brochure

Current international guidelines promote exclusive breastfeeding for six months by all HIV negative women, women of unknown status and HIV positive women who either choose to breastfeeding and/or do not meet the AFASS criteria for replacement feeding [2]. A major concern in the development of the integrated set of materials was the need for a breastfeeding brochure that was 'universally acceptable', that could be used as an educational and promotional tool with the general population. Consequently, the team took great caution in developing the brochure to: 1) support efforts to promote exclusive breastfeeding for the first six months of age; 2) avoid any association between exclusive breastfeeding and HIV positive status; and 3) ensure that HIV positive mothers using the brochure were not "exposed" or inadvertently put in jeopardy.

**Figure 2:**
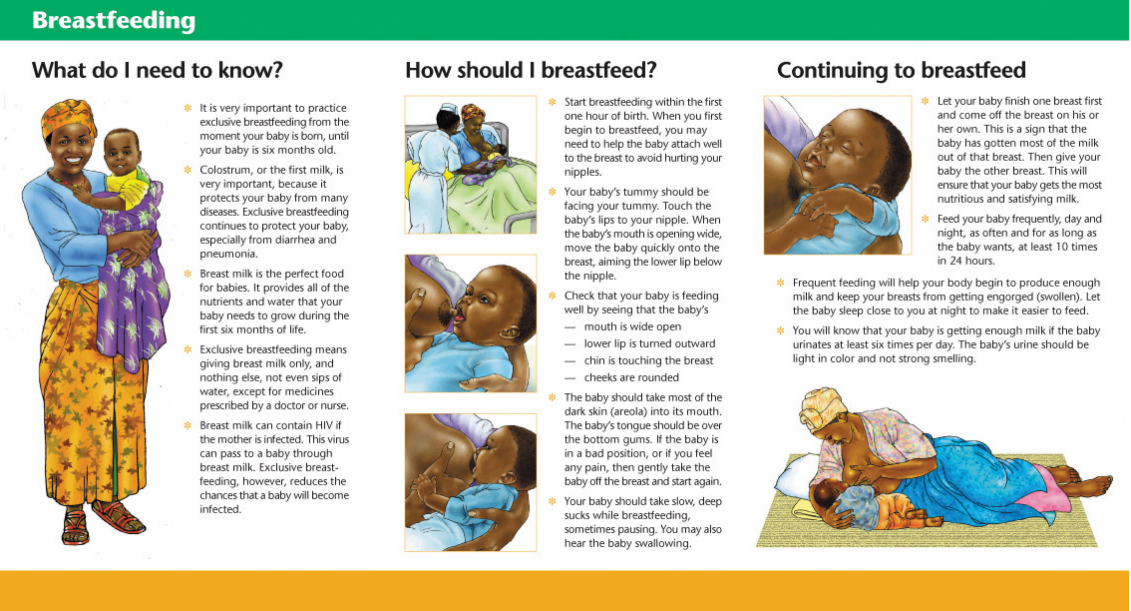
Shows how to breastfeed a baby.

**Figure 3:**
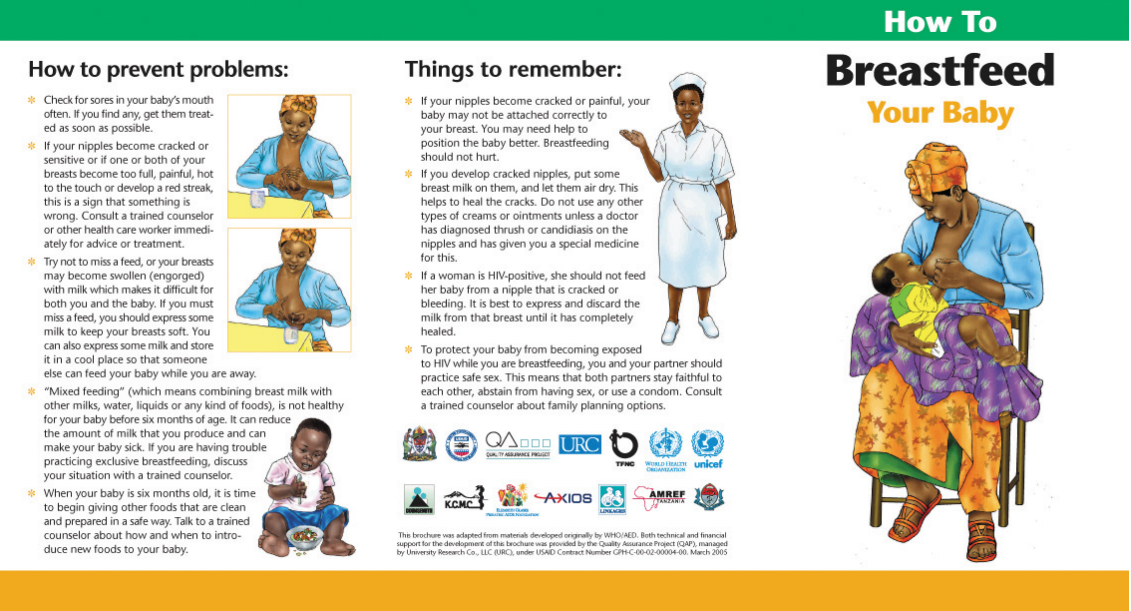
Shows how to prevent breast problems when breastfeeding.

Unlike the other materials, the breastfeeding brochure was specifically designed to be used in counselling all prenatal or postpartum women – HIV positive, HIV negative and women of unknown status through pMTCT programmes as well as antenatal, postpartum and well child clinics. Strategically, the brochure does not refer to HIV status. The cover features a culturally sensitive image of a Tanzanian mother breastfeeding her baby. The text and illustrations emphasise the importance of *exclusive *breastfeeding on demand and the avoidance of water or any other liquids or solid foods during the first six months of life. The images illustrate proper positioning and attachment to reduce breast pathology (such as engorgement, soreness, bleeding and abscesses), how to cope with common *breastfeeding problems *and the importance of practising *safe sex *with emphasis on using a condom, especially while breastfeeding. (See Figures 2 and 3.)

#### Replacement feeding brochures

**Figure 4:**
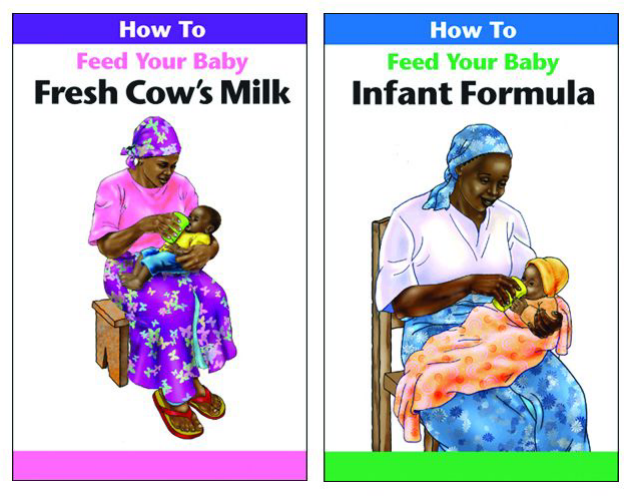
Shows how to feed infant formula and modified cow's milk to the baby.

Two brochures addressing replacement feeding options (cow's milk, infant formula) each portray an image on the cover of a mother feeding her baby using a cup rather than a bottle. The images and the text of the cow's milk brochure emphasise the use of local resources (utensils and wood fires); safe procedures for the preparation of the milk; and the steps needed to calculate and mix the appropriate quantities of milk, water, sugar and micronutrients for each feed according to the baby's age. Similarly, the brochure on infant formula illustrates safe procedures for preparing utensils, boiling the water; and calculating the right amounts of formula powder and water for each feed, according to the baby's age. Both brochures emphasise using an open cup to feed the baby, avoiding mixed feeding, the importance of safe sex, and the use of family planning to achieve adequate child spacing. (See Figure 4.)

#### Expression and heat treatment brochure

Given the cost and other AFASS issues associated with replacement feeding, the expression and heat treatment of breast milk was included as a possible feeding option in the updated international guidelines. The effect of heat treatment in reducing the risks associated with breastfeeding related HIV transmission has been documented [31,32], and its feasibility and acceptability, especially during the transition from exclusive breastfeeding to exclusive replacement feeding, have been demonstrated in several settings in sub-Saharan Africa [33,34]. Discussions around expression and heat treatment throughout the present study, however, revealed a split of interests between the international technical actors (WHO, UNICEF and research institutions) and local stakeholders (counsellors, mothers and community members). Because the initial reaction of study participants in Moshi to both expressing and heating breast milk was undeniably negative, the decision to include a brochure on this method as part of the intervention deserves a special note. With the intent of exploring issues related to heat treatment and positioning this method for possible use in the future, formative research findings were used to improve the draft illustrations and ensure that the content was as clear and visually appealing as possible. Due to the underlying client centred philosophy of the intervention, however, this brochure was presented to counsellors during their one day training, but was not actively promoted as a feeding option during counselling conducted under the subsequent operations research study at KCMC.

#### The counselling card on relative risk

**Figure 5:**
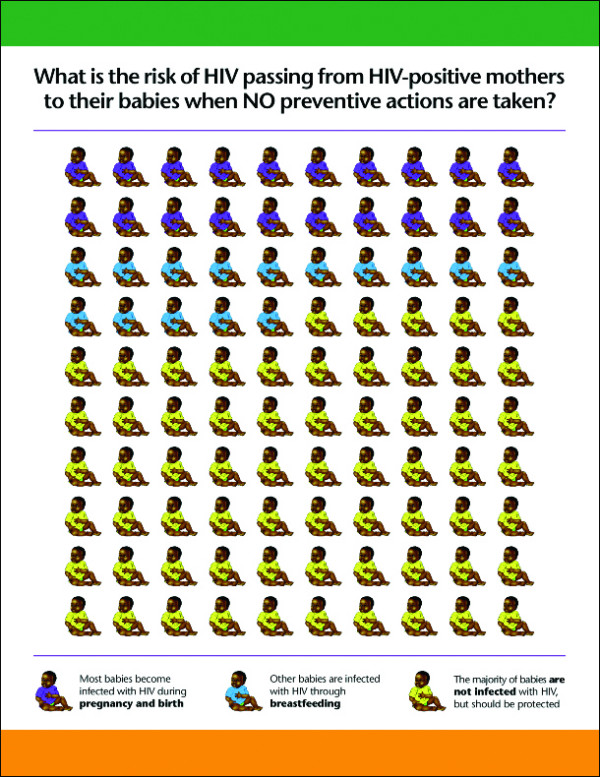
Shows counselling card on relative risk of HIV transmission from mother to child when there are no preventive measures used.

The counselling card explains the relative risk of HIV transmission from mother to child, based on a WHO generic counselling material. The card graphically presents the number of babies infected during pregnancy, birth and breastfeeding from among 100 babies born to HIV infected mothers. This graphic design was based on the mothers' level of literacy to communicate at both their emotional and cognitive level using something they can easily identify. (See Figure 5.)

#### The infant feeding 'tool box'

**Figure 6:**
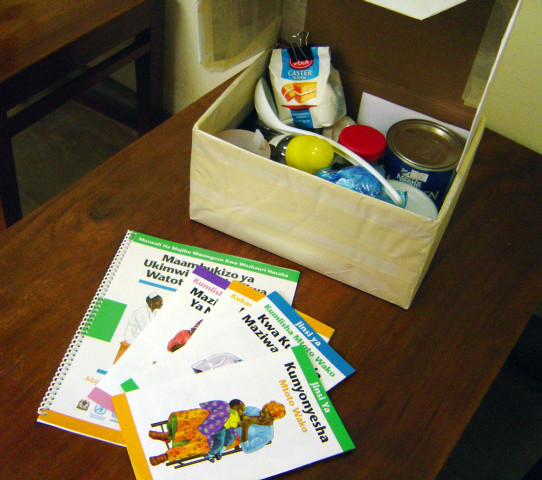
Shows the infant feeding tool box for demonstration during counselling session.

The infant feeding tool box was designed to be used in counselling sessions and contains basic items such as cups, spoons, sample tin of formula, thermos, pot, sugar and micronutrients needed to demonstrate how to prepare infant formula and cow's milk respectively. It also contained soap for washing hands and cleaning utensils. (See Figure 6.)

### The technical review process and incorporation of technical feedback

After field testing the draft illustrations at the community level, the modified illustrations were incorporated into the layout of key text messages for each material. Electronic versions (PDFs) of the job aids (both in English and Swahili) were widely circulated by email for technical review by local and national stakeholders and other national and international technical experts. Comments were incorporated and adjustments made to the technical content and illustrations prior to producing a limited package of the integrated set for use in a one day training/orientation workshop for 15 nurse counsellors from the KCMC pMTCT Programme. During this event, additional technical comments and corrections to both the English and Swahili translations were received and incorporated. All changes were made prior to printing a sufficient quantity for use during the six month operations research study to assess the strengths and weaknesses of the job aids, to be reported in a forthcoming article. The significance of the one day training/orientation workshop, which focused on interpersonal communication, counselling skills and the effective use of the job aids, is also reported elsewhere.

## Conclusion

This study recognizes that infant feeding norms and practices are produced and reproduced or transformed in the encounter between local ideas and customs on the one hand and forces emanating from the larger national and international community on the other. Through participatory qualitative research, this study aimed to adapt the international WHO/UNICEF guidelines on HIV and infant feeding and related generic counselling tools to the local social and cultural context of infant feeding and HIV in the Kilimanjaro Region of northern Tanzania. Because infant feeding practices are socially and culturally embedded, community norms and the cultural beliefs and practices of mothers and those who influence them must be taken into consideration in designing an intervention. Tailoring the present educational intervention to the specific needs and characteristics of the study participants helped to ensure that this intervention would be socially and culturally acceptable to the targeted study population, and underscores the importance of formative research in the intervention development process.

Although the utility of applying theoretical frameworks to the design and execution of interventions has been questioned [35], IM provided a useful reference to guide the development of the educational material (job aids) presented in this study, through a dual focus on health promotion theory and empirical evidence obtained through formative research,. Given restrictions on time and other resources, a modified version of IM was applied, which restricted the complexity of the change objectives. Nonetheless, IM was a valuable tool in the development of objectives, methods, strategies, materials and procedures. Through the definition of performance objectives, modifiable determinants and specified learning objectives as outlined in Tables 1 and 2, outcome indicators were identified at both the individual and environmental levels.

To ensure "ownership" of or "buy in" to the intervention by key stakeholders and to position this pilot intervention for subsequent scale up, the development process required the active and strategic participation of all relevant stakeholders, including participation in the initial review of the intervention strategy and technical reviews of the related products. Through the participatory approach prescribed, IM facilitated an active and systematic dialogue with all relevant actors.

Through the needs assessment, the intervention planning and the strategy and job aids development process, a number of questions related to potential impact and sustainability of the intervention emerged. As the IM framework underscores, change is very often the result of change in the behaviour of decision makers and key actors on multiple levels. For example, as documented in the current paper, there is no doubt that a woman's husband, her mother in law and her pMTCT counsellor are important actors in her infant feeding environment. pMTCT as a family issue remains tricky, in particular because of challenges related to the issues of confidentiality and disclosure. The framework reported in the current intervention primarily addresses individual motivational factors, i.e. factors internal to the mother herself. Changing knowledge, attitudes and beliefs is critical for behavioural change but may not be sufficient to change mothers' infant feeding practices. There are many factors, barriers and facilitators of change, that contribute to mothers' decisions concerning the feeding of their babies. These factors vary depending on determinants of choice and the individual or group of mothers in question.

In the context of HIV, stigma and the fear of disclosure of positive HIV status is a major concern influencing mothers' infant feeding choice, even though they are highly committed to preventing the transmission of HIV to their babies. This study underscores the complexity of promoting recommended infant feeding practices and clearly indicates the need for a multi-dimensional behaviour change strategy involving both mothers and counsellors, and if possible significant others who influence decision making processes. In a context where disclosure of status is a major challenge, the participation of partners and other relatives in counselling, although ideal, is seldom realized.

'Informed choice' related to infant feeding in the context of HIV/AIDS is a complex issue. Access to information and improved interpersonal communication and counselling are among many factors influencing an HIV positive mother's confidence, courage and ability to select and successfully implement the most appropriate feeding option given her own individual situation. This intervention study underscores the importance of providing culturally compatible counselling support that improves self-esteem and confidence and corresponds with the social norms and perceptions of mothers.

## Competing interests

The author(s) declare that they have no competing interest.

## Authors' contributions

SCL, PKB and KMM contributed to conception and design of the study, analysis and interpretation of data, revised drafts of job aids, illustrations and key messages. Also drafted and revised the manuscript drafts. PKB managed the developed and external technical review of the draft job aids. MDP revised drafts of job aids illustrations and key messages and revised manuscript drafts. ANA critically revised the manuscript drafts and gave advice on the theoretical approach. All authors revised and approved the last version of the manuscript.

## References

[B1] Thairu LN, Pelto GH, Rollins NC, Bland RM, Ntchangase N (2005). Sociocultural influences on infant feeding decisions among HIV-infected women in rural Kwa-Zulu Natal, South Africa. Maternal and Child Nutrition.

[B2] WHO (2003). WHO/UNICEF/UNFPA/UNAIDS. HIV and Infant Feeding: Guidelines  for Decision-makers. Reviewed.

[B3] Kuhn L, Stein Z, Susser M (2004). Preventing mother-to-child transmission in the new millennium: the challenge of breast feeding. Paediatric and Perinatal Epidemiology.

[B4] DeKock KM, Fowler MG, Mercier E, de Vincenzi I, Saba J, Hoff E (2000). Prevention of mother-to-child HIV transmission in resource poor countries. Journal of the American Medical Association.

[B5] UNAIDS (2005). Global Summary of the AIDS epidemic. AIDS epidemic update, December.

[B6] Latham MC, Preble E (2000). Appropriate feeding methods for infants of HIV infected mothers in sub-Saharan Africa. British Medical Journal.

[B7] Iliff PJ, Piwoz EG, Tavengwa NV, Zunguza CD, Marinda ET, Nathoo KJ, Moulton LH, Ward BJ, Humphrey JH (2005). Early exclusive breast-feeding reduces the risk of postnatal HIV-1 transmission and increases HIV-free survival. AIDS.

[B8] Coutsoudis A, Pillay K, Kuhn L, Spooner E, Tsai WY, Coovadia HM (2001). Method of feeding and transmission of HIV-1 from mothers to children by 15 months of age: prospective cohort study from Durban, South Africa. Aids.

[B9] Coutsoudis A, Pillay K, Spooner E, Kuhn L, Coovadia HM (1999). Influence of infant-feeding patterns on early mother-to-child transmission of HIV-1 in Durban, South Africa: a prospective cohort study. South African Vitamin A Study Group. Lancet.

[B10] Coutinho SB, de Lira PI, de Carvalho Lima M, Ashworth A (2005). Comparison of the effect of two systems for the promotion of exclusive breastfeeding. Lancet.

[B11] Manongi R, Klepp KI, de Paoli (2002). Counsellors' perspectives on antenatal HIV testing and infant feeding dilemmas facing women with HIV in northern Tanzania. Reproductive Health Matters.

[B12] Bland RM, Thairu L, Coovadia HM, P. Koniz-Booher BBAWPIJW, Rollins (2002). Counselling HIV-infected women on infant feeding choices in rural South Africa.. A Compilation of Programmatic Evidence, 2004 (pp 54-55) USAID, UNICEF & QAP-URC (full text: wwwhivgovgy/edocs/compilation_hivinfantfeedingpdf).

[B13] Nduati R, John G, Mbori-Ngacha D, Richardson B, Overbaugh J, Mwatha A, Ndinya-Achola J, Bwayo J, Onyango FE, Hughes J, Kreiss J (2000). Effect of breastfeeding and formula feeding on transmission of HIV-1: a randomized clinical trial. Jama.

[B14] Kiarie JN, Richardson BA, Mbori-Ngacha D, Nduati RW, John-Stewart GC (2004). Infant feeding practices of women in a perinatal HIV-1 prevention study in Nairobi, Kenya. J Acquir Immune Defic Syndr.

[B15] Chopra M, Doherty T, Jackson D, Ashworth A (2005). Preventing HIV transmission to children: quality of counselling of mothers in South Africa. Acta Paediatr.

[B16] Koniz-Booher PBBWAIPWE (2004). HIV and infant feeding A compilation of programmatic evidence, Published for UNICEF and USAID by the Quality Assurance Project, University Research Co., LLC, Bethesda, MD. (full text: www.hiv.gov.gy/edocs/compilation_hivinfantfeeding.pdf).

[B17] Swartzendruber A, Msamanga G, Team PMTCTE (2002). Programme Report: "Evaluation of the UNICEF-Sponsored Prevention of Mother-to-Child HIV Transmission (PMTCT) Pilot Sites in Tanzania".

[B18] Ehrnst A, Zetterstrom R (2005). Feeding practices of HIV-1-infected mothers: the role of counsellors. Acta Paediatr.

[B19] Kok G, Schaalma H, Ruiter RA, Empelen PV (2004). Intervention mapping: protocol for applying health psychology theory to prevention programmes. Journal of Health Psychology.

[B20] Kim YM, Kols A, Martin A, Silva D, Rinehart W, Prammawat S, Johnson S, Church K (2005). Promoting Informed Choice: Evaluating A Decision-Making Tool for Family Planning Clients And Providers in Mexico. International Family Planning Perspectives.

[B21] Knebel E, Lundahl S, Edward RA, Abdallah H (2000). The Use of Manual Job Aids by Health Care Providers: What do we know? Operations research issue paper 1 (1).

[B22] O'Connor AM, Stacey D, Rovner D, Holmes-Rovner M, Tetroe J, Llewellyn-Thomas H, Entwistle V, Rostom A, Fiset V, Barry M, Jones J (2001). Decision aids for people facing health treatment or screening decisions. Cochrane Database Syst Rev.

[B23] Ministry of Health (2005). National AIDS Control Programme, Surveillance of HIV and Syphilis Infections Among Antenatal Clinic Attendees 2003/04.

[B24] Green LW, Kreuter MW (1999). Health promotion and planning: An educational and ecological approach, 3rd edn..

[B25] Bartholomew K, Parcel G, Kok G, Gottlieb N (2001). Intervention Mapping: Developing theory-and evidence-based health education programs.

[B26] Bandura A (1986). Social Foundations of Thought and Action: A Social Cognitive Theory.

[B27] Contento IR, Michela JL, Williams SS (1995). Adolescent food choice criteria: role of weight and dieting status. Appetite.

[B28] Burnard P (1991). A method of analysing interview transcripts in qualitative research. Nurse Education Today.

[B29] Kvale S (1996). Interviews: An introduction to qualitative research interviewing.

[B30] Kim YM, Putjuk A, Kols A, Basuki E (2000). Improving provider-client communication: Reinforcing IPC/C training in Indonesia with self-assessment and peer review. Operations Research Results 1 (6). Published for the United States Agency for International Development (USAID) by the Quality Assurance Project (QAP):Bethesda, Maryland..

[B31] Chantry CJ, Morrison P, Panchula J, Rivera C, Hillyer G, Zorilla C, Diaz C (2000). Effects of lipolysis or heat treatment on HIV-1 provirus in breast milk. J Acquir Immune Defic Syndr.

[B32] Israel-Ballard K, Chantry C, Dewey K, Lonnerdal B, Sheppard H, Donovan R, Carlson J, Sage A, Abrams B (2005). Viral, nutritional, and bacterial safety of flash-heated and pretoria-pasteurized breast milk to prevent mother-to-child transmission of HIV in resource-poor countries: a pilot study. J Acquir Immune Defic Syndr.

[B33] Israel-Ballard KA, Maternowska MC, Abrams BF, Morrison P, Chitibura L, Chipato T, Chirenje ZM, Padian NS, Chantry CJ (2006). Acceptability of heat treating breast milk to prevent mother-to-child transmission of human immunodeficiency virus in Zimbabwe: a qualitative study. J Hum Lact.

[B34] Jeffery BS, Mercer KG (2000). Pretoria pasteurisation: a potential method for the reduction of postnatal mother to child transmission of the human immunodeficiency virus. J Trop Pediatr.

[B35] Oxman AD (2005). Variance and dissent. The OFF theory of research utilization. Clinical Epidemiology.

